# Implicit Maternal Intuition Confidence Is Associated With Maternal Well-Being Across Cultures

**DOI:** 10.3389/fpsyg.2020.00289

**Published:** 2020-02-21

**Authors:** Wendi L. Gardner, Katie N. Rotella, Janeta Nikolovski

**Affiliations:** ^1^Department of Psychology, Northwestern University, Evanston, IL, United States; ^2^Johnson & Johnson Consumer, Inc., Skillman, NJ, United States

**Keywords:** implicit measures, parenting, intuition, well-being, culture

## Abstract

The transition to motherhood involves the experience of each individual mother and child, as well as the burden of cultural expectations. Social desirability demands may impede self reports of difficulties during the transition to motherhood when using traditional explicit measures. One core component of maternal role attainment is a mother’s confidence in her own intuitive knowledge of her child. This brief report presents two studies that examine a “low technology” implicit measure of maternal intuition confidence that is based within a more general decision confidence paradigm. Study 1 examined the association of both implicit and explicit maternal intuition confidence with depressive symptoms, life satisfaction, and maternal identity satisfaction in a United States sample of mothers. The implicit measure contributed to variance in each of the outcome measures, above and beyond an explicit measure. Study 2 explored the association of implicit maternal intuition confidence with life satisfaction and maternal identity satisfaction in Brazil, China, India, the United States and the United Kingdom. Across all samples, implicit maternal intuition confidence was significantly associated with satisfaction with life. However, it was significantly associated with maternal identity satisfaction only in the two individualistic countries (the United States and the United Kingdom), but not in the three collectivist countries.

## Introduction

The capacity of implicit measures to tap thoughts or feelings that yield distinct information from explicit questioning has been a boon for research on difficult topics ranging from racial prejudice to suicidality (Sleek, 2018). The majority of implicit measures rely on the assessment of computerized reaction time under cognitive conflict (IAT, Greenwald and Banaji, 1995) or after priming (AMP, Payne et al., 2005), but physiological assessments such as facial EMG (Roddy et al., 2010) and the startle blink response (Mahaffey et al., 2011) have also proven valuable.

Not all researchers who could benefit from utilizing implicit measures may have the opportunity, resources, or expertise to use technologically advanced procedures, however. The current research explores a “low technology” procedure, one that capitalizes on the experiential processes driving decision confidence under uncertainty (Schwartz, 2004). We examine the utility of this procedure to serve as an implicit measure in a domain fraught with social desirability concerns, that of the transition to motherhood.

Mothers experience strong societal pressure to exemplify maternal “ideals,” including unceasing warmth, omnipotent understanding, and continuous responsiveness to their infant (Hays, 1996). For example, in one survey of postpartum bonding (Wittkowski et al., 2007) zero out of ninety-six mothers self-reported ever feeling “resentful” of their infant, perhaps implying self-censorship of explicit responses that conflict with cultural ideals. Indeed, social desirability biases in mothers’ explicit self-reports emerge during pregnancy (van Bussel et al., 2010) and last through their child’s adolescence (Nederhof, 1985).

Implicit measures may thus be especially valuable when applied to self-assessments of maternal feelings and beliefs. Moreover, because research on maternal well-being is often conducted during “well-baby” visits to the pediatrician’s office or home visits in early childhood intervention studies, an ideal index would sidestep social desirability concerns without requiring sophisticated measurement technology.

The current work focused on a form of maternal self-assessment especially conducive to implicit measurement, that of confidence in maternal intuition. One of the greatest challenges during the transition to motherhood is learning to understand the unique signals of an infant. In the classic model of “becoming a mother” (Mercer, 2004), feelings of competence in knowing the baby’s feelings and preferences (maternal intuition) are critical to both maternal well-being and the attainment of a satisfying maternal identity. Confidence in intuitive understanding of one’s infant is distinct from confidence in effective parenting behaviors, yet it is the intuitive understanding that is almost universally perceived as being central to motherhood. Mesman et al. (2015) found striking convergence across twenty-six cultural groups that both accurate understanding and sensitive responsiveness to the child were core components of the “ideal mother.” Given theoretical importance to maternal well-being and identity, in addition to vulnerability to social desirability concerns, an *implicit* assessment of maternal intuition confidence seems especially valuable.

Fortunately, several “low tech” implicit measurement techniques exist (see Sekaquaptewa et al., 2010, for review), including techniques designed to assess confidence in intuitive judgments. Some judgments (e.g. “What percentage of days in July are above 85 degrees?”) require one’s “best guess” based on limited or uncertain explicit knowledge. *Confidence* in that judgment then reflects both the feelings of confidence experienced when making the decision, and beliefs concerning one’s own competence in the domain (Schwartz, 2004). Kamat (2011) capitalized on this finding to develop a measure of “general intuition confidence” designed to tap implicit feelings of self-certainty. In a two-part procedure, participants are first asked a question for which explicit knowledge of the correct answer is unavailable (e.g. “What percentage of Americans do you think would prefer to travel to Italy for vacation over Spain?”), forcing them to give the answer that intuitively feels right. Second, participants report confidence in that decision. This procedure, repeated over several decisions and confidence judgments, assesses a participants general confidence in their intuition. Hamilton et al. (2011), using a similar two-step procedure, demonstrated that decision confidence measures (but not the decisions themselves) were influenced by experimental manipulations of identity confusion. The current work applies the two-step procedure (Kamat, 2011), specifically to the domain of “maternal intuition confidence.” Each of the initial decisions require intuitive knowledge of one’s own infant (e.g. “When your baby cries, what percentage of the time is it due to physical discomfort versus psychological distress”), mothers then rate their confidence in each decision.

The aim of this research was to examine the use of this decision confidence assessment as a potential implicit measure of maternal intuition confidence. Because prior work proposed that maternal intuition confidence was a cornerstone to subjective maternal satisfaction and maternal identity development (Mercer, 2004), we examined the associations of this measure with those outcomes. We also compared those associations with an explicit assessment of maternal intuition confidence (Study 1), and examined potential cultural differences in the relationship of implicit maternal intuition confidence to life satisfaction and maternal identity by sampling mothers from five different countries (Study 2).

## Study 1

Study 1 explored whether an implicit confidence-in-intuition measure used in previous studies to assess general decision confidence could be applied to the more specialized context of maternal intuition confidence. We developed a measure that tapped mothers feelings of confidence in their decisions concerning knowledge of their baby’s needs/preferences in four different childcare situations. We also assessed implicit general intuition confidence and explicit parental intuition confidence to explore the relationship among the three measures, as well as to test the hypothesis that the implicit measure of maternal intuition confidence would hold unique value in explaining variability in depressive symptoms, satisfaction with life, and maternal identity.

### Method

#### Participants

Two hundred and seventy-two North American mothers of babies under the age of 24 months were recruited to take an online survey using Amazon’s Mechanical Turk platform. Age of mothers ranged from 18 to 44 (*M* = 28). Seventy percent of participants identified as European American, with 12% identifying as Black, 12% as LatinX or Hispanic, and 6% as Asian American. The sample was roughly equal in new and experienced mothers, with 51% reporting being first-time mothers, 37% reporting having one other child, and 11% reporting having two or more additional children. Eighteen percent of mothers reported their baby was under the age of 6 months, 41% between 6 and 12 months, and 41% between 12 and 24 months.

#### Materials

All materials for this study may be accessed at https://osf.io/38s6t/

##### Implicit General Intuition Confidence

A two-part procedure (Kamat, 2011) for each of two items first presented participants with a question that participants would have no knowledge of the correct answer, thus must give the answer that intuitively feels right (e.g. “What percentage of Americans do you think would prefer to travel to Italy for vacation over Spain?” 1 = *100% Italy;* 2 = *90% Italy, 10% Spain*…11 = *100% Spain*). After each, participants then answer “How confident are you in your prior answer?” 1 = *Not at all* to 7 = *Extremely.* Confidence scores are averaged to create the index of implicit general intuition confidence (*M* = 3.99, *SD* = 1.44).

##### Implicit Maternal Intuition Confidence

We adapted the general intuition confidence procedure to be specific to parenting, using 4 two-part responses. In each response mothers were first asked a question concerning understanding their baby in one of four key situations (crying, feeding, sleep, and play – e.g. “When your baby cries, what percentage of the time is it due to physical discomfort versus psychological distress” 1 = *100% of crying is due to physical discomfort;* 2 = *90% physical, 10% psychological*…11 = *100% of the time is due to psychological distress*.). Each item was immediately followed by asking “How confident are you in your prior answer?” 1 = *Not at all* to 7 = *Extremely.* The four “confidence” responses were averaged to create the index of implicit maternal intuition confidence (*M* = 5.62, *SD* = 0.78).

##### Explicit Maternal Intuition Confidence

Adapted from the Parental Self Efficacy Scale (Gibaud-Wallston and Wandersman, 1978) e.g. “If anyone can find the answer to what is troubling my baby, I am the one” 1 = *Strongly disagree*; 6 = *Strongly agree* (*M* = 4.88, *SD* = 1.03).

##### Depressive Symptoms

Ten-item Center for Epidemiologic Studies Short Depression Scale. e.g. “In the past week I felt depressed.” 0 = *Not at all* to 4 = *Nearly every day.* Scores are sums (*M* = 16.99, *SD* = 5.33). The item “My sleep was disrupted” was not used in the index, given sleep disruption is common and non-diagnostic in new parents.

##### Satisfaction with Life

Five-item scale (Diener et al., 1985). e.g. “In most ways, my life is close to ideal.” 1 = *Strongly Disagree* to 7 = *Strongly Agree* (*M* = 5.19, *SD* = 1.29).

##### Maternal Identity Satisfaction

Adapted from Sellers et al. (1998). e.g. “Overall, being a mother is the most fulfilling aspect of my life” 1 = *Not at all true of me* to 7 = *Extremely true of me* (*M* = 5.87, *SD* = 1.22).

#### Procedure

After written informed consent, participants completed a survey including the measures listed above. The implicit maternal intuition confidence assessment was presented in counterbalanced order with the explicit maternal intuition confidence and implicit general intuition confidence measures. Satisfaction with life, depressive symptomology, and maternal identity satisfaction were then presented in counterbalanced order.

### Results and Discussion

Hallmarks of implicit measures include (1) low correlations with their explicit counterparts, (2) distinct antecedents and/or consequences from explicit measures, explaining unique variance in associated outcomes. We first examined the correlation between the implicit and explicit maternal intuition measures, and then examined whether and how the experience of the mother (first time vs. experienced mother) and the stage of the baby (under 6 mos, 6–12 mos, 12–24 mos) were related to each. Finally, we examined whether implicit maternal intuition confidence would show unique associations with subjective well-being as well as with maternal identity satisfaction.

Implicit and explicit measures of maternal intuition confidence showed a small but significant correlation, *r* (272) = 0.22, *p* < 0.001., implying they were related, but non-redundant. Additionally, and as expected, implicit maternal intuition confidence was correlated with implicit general intuition confidence, *r* (272) = 0.23, *p* < 0.001, whereas explicit maternal intuition confidence was unrelated to implicit general intuition confidence, *r* (272) = −0.03, *p* = 0.62.

Prior research has revealed that the transition to motherhood is equally difficult for first and subsequent babies (e.g. Krieg, 2007), in part because each individual infant brings unique challenges. Qualitative studies of maternal intuition confidence also suggest that a mother’s intuitive confidence in understanding her baby’s signals grows over time with that individual baby (e.g. Mercer, 2004). Analyses examining whether prior experience as a mother influenced implicit maternal intuition confidence were conducted using a two Experience (first-time mother; experienced mother) ANCOVA with implicit general intuition confidence as a covariate in order to focus solely on maternal intuition confidence. No relationship was found with prior experience as a mother and implicit maternal intuition confidence (*M*_new_ = 5.61; *SD* = 0.79, *M*_experienced_ = 5.63, *SD* = 0.77, *p* = 0.84), Explicit maternal intuition confidence also did not differ as a function of maternal experience (*M*_new_ = 4.94, *SD* = 1.01; *M*_experienced_ = 4.81, *SD* = 1.05; *p* = 0.31). However, a three Baby-age (<6 mos; 6–12 mos; 12–24 mos) ANCOVA with implicit general intuition confidence as a covariate supported the notion that more time with the baby is important and/or that older babies are easier to understand, as implicit maternal intuition confidence significantly increased across baby-age (*M*s = 5.40, 5.52, 5.82; *SD*s = 0.82, 0.74, 0.77) *F* (2, 271) = 7.06, *p* < 0.001. Interestingly, an ANOVA examining explicit intuition confidence did not show similar effects (*M*s = 4.81, 4.87, 4.90; *SD*s = 1.06, 1.06, 1.01) *F* < 1, *p* = 0.88.

We conducted three linear regression analyses predicting depressive symptomology, satisfaction with life, and maternal identity satisfaction from implicit and explicit maternal intuition confidence. For each of these analyses, implicit general intuition confidence was entered on the first step, then implicit and explicit intuition confidence entered simultaneously in the second step. As Table 1 highlights, implicit maternal intuition confidence was uniquely and significantly associated with all three outcome variables, over and above the contributions of explicit maternal intuition confidence. Explicit maternal intuition confidence was significantly related to depressive symptomology and maternal identity satisfaction and was positively but non-significantly related to satisfaction with life. Taken in combination, the results of Study 1 reveal that implicit and explicit maternal intuition confidence were only modestly correlated, that implicit but not explicit maternal intuition confidence was sensitive to baby-age, and that each carried unique explanatory variance in understanding well-being as assessed by both depressive symptoms and satisfaction with life, as well as in maternal identity satisfaction.

**TABLE 1:** Implicit and explicit maternal intuition confidence as predictors of maternal well-being and satisfaction, controlling for implicit general intuition confidence.

	**Depressive symptomology**	**Satisfaction with life**	**Maternal identity satisfaction**
Implicit maternal intuition confidence	β = −0.13, *t* (268) = −2.09, *p* = 0.037	β = 0.18, *t* (268) = 2.89, *p* = 0.004	β = 0.16, *t* (268) = 2.63, *p* = 0.009
Explicit maternal intuition confidence	β = −0.14, *t* (268) = −2.29, *p* = 0.023	β = 0.09, *t* (268) = 1.57, *p* = 0.12	β = 0.33, t (268) = 5.71, *p* = 0.000

## Study 2

Study 2 provided a replication and extension of Study 1. We had the opportunity to include the measures of implicit maternal and general intuition confidence as well as satisfaction with life and maternal identity satisfaction in a large survey examining childcare practices in first-time mothers across five cultures (Brazil, China, India, the United Kingdom, and the United States). The United Kingdom and United States samples provide replication opportunities for the associations of implicit maternal intuition confidence in the well-being and identity measures found in United States mothers in Study 1, as both the United States and the United Kingdom represent individualist countries. Because Brazil, India, and China are all considered collectivist countries (Triandis, 1995), these samples provide a valuable extension to assess whether implicit maternal intuition confidence is similarly associated with maternal satisfaction and identity across both individualist and collectivist countries.

Competing hypotheses exist for cultural specificity vs. universality of the relationship between implicit intuition confidence and maternal well-being. Prior research has shown that internal affective information is less important to overall life satisfaction in collectivist countries (e.g. Suh et al., 1998), implying that implicit maternal intuition may not be as strongly associated with well-being in collectivist as compared to individualist countries. On the other hand, the universality of both the need to understand non-verbal infants and of the perception that accurate responsiveness toward one’s baby reflects ideal motherhood (Mesman et al., 2015) may suggest that high maternal intuition confidence would be similarly beneficial for life satisfaction across countries.

Hypotheses are clearer for the potential role of intuition confidence in maternal identity. Because individualist versus collectivist countries differ in the way in which identity is defined as either reflecting internal traits and feelings versus externally validated roles in relationships and groups (Markus and Kitayama, 1991; Gardner et al., 1999), implicit maternal intuition confidence may be more strongly related to maternal identity satisfaction in individualist than collectivist countries.

### Method

#### Participants

Seven hundred and forty-five mothers were recruited for a paid survey through HCD Research, Inc. One hundred forty-three mothers participated from Brazil, 151 from China, 151 from India, 150 from the United Kingdom, and 150 from the United States. All participants were first-time mothers of infants under 6 months of age and were all themselves under the age of 35. Participants were prescreened for post-partum depression, and only non-depressed mothers were invited to participate.

#### Materials

Implicit general intuition confidence and implicit maternal intuition confidence, satisfaction with life, and identity satisfaction were identical to the measures in Study 1, with the exception that the initial implicit general intuition measure questions were changed to be appropriate to an international sample (e.g. “What percentage of *mothers in your country* do you think would prefer to travel to Italy for vacation over Spain?”). All materials were translated into the participants’ language in a multi-step translation and editing process involving two native speakers (for each language) providing independent translations that were then checked for consistency (or corrected) by a third native speaker before the translated survey was finalized.

#### Procedure

After being prescreened for eligibility and completing written informed consent, participants completed the items above as part of a larger survey on childcare experiences.

### Results and Discussion

Table 2 provides descriptive data from each country. Brazil, China, and India represent collectivist countries, and the United Kingdom and the United States represent individualist countries. To examine whether cultural differences in implicit maternal intuition confidence emerged, a two Country type (individualist, collectivist) ANCOVA with implicit general intuition confidence as a covariate was conducted. Mothers from individualistic countries had significantly lower implicit maternal intuition confidence scores than mothers from collectivist countries (*M*_ind_ = 5.48, *SD* = 0.89; *M*_coll_ = 5.82, *SD* = 0.71), *F* (1,742) = 33.86, *p* < 0.001.

**TABLE 2:** Descriptive statistics by country.

	**Implicit maternal intuition confidence**	**Implicit general intuition confidence**	**Satisfaction with life**	**Maternal identity satisfaction**
Brazil	*M* = 5.80, *SD* = 0.81	*M* = 5.21, *SD* = 1.24	*M* = 5.35, *SD* = 1.16	*M* = 5.86, *SD* = 1.24
China	*M* = 6.00, *SD* = 0.63	*M* = 5.85, *SD* = 0.78	*M* = 5.26, *SD* = 0.96	*M* = 3.17, *SD* = 1.87
India	*M* = 5.94, *SD* = 0.65	*M* = 5.88, *SD* = 0.88	*M* = 6.08, SD = 0.61	*M* = 5.67, *SD* = 1.25
United Kingdom	*M* = 5.26, *SD* = 0.89	*M* = 4.76, *SD* = 1.42	*M* = 5.05, SD = 0.96	*M* = 5.44, *SD* = 1.35
United States	*M* = 5.42, *SD* = 0.83	*M* = 4.72, *SD* = 1.42	*M* = 5.37, *SD* = 1.09	*M* = 5.59, *SD* = 1.50

Our core hypothesis concerned whether implicit maternal intuition confidence was differentially associated with satisfaction with life and maternal identity satisfaction in individualist versus collectivist countries. For these analyses, individualist-collectivist country type was entered in the first step, along with implicit general intuition confidence and the interaction term, maternal intuition confidence and its interaction with individualist-collectivist country type were then entered simultaneously in the second step. Implicit maternal intuition confidence was positively associated with satisfaction with life and was not qualified by an interaction with culture type. Follow-up analyses revealed that implicit maternal intuition confidence was positively and significantly associated with satisfaction with life (*β*s ranging from 0.21 in the United Kingdom to 0.35 in China) in each of the five nations studied. In contrast, an interaction between implicit maternal intuition confidence and culture type emerged for identity satisfaction, *t* (739) = 2.85, *p* = 0.005. As Table 3 illustrates, the positive association between implicit maternal intuition confidence and maternal identity satisfaction replicated in individualist countries; no significant association emerged in collectivist countries.

**TABLE 3:** Implicit maternal intuition confidence as a predictor of maternal well-being and satisfaction in individualist and collectivist countries, controlling for implicit general intuition confidence.

	**Satisfaction with life**	**Maternal identity satisfaction**
**INDIVIDUALIST**		
Implicit maternal intuition confidence	β = 0.29, *t* (297) = 4.95, *p* = 0.000	β = 0.28, *t* (297) = 5.01, *p* = 0.000
**COLLECTIVIST**		
Implicit maternal intuition confidence	β = 0.21, *t* (442) = 3.90, *p* = 0.000	β = –0.02, *t* (442) = −0.41, *p* = 0.684

## General Discussion

These two studies demonstrated that a “low technology” implicit measure of maternal intuition confidence was reliably associated with aspects of maternal well-being. Results from Study 1 with mothers from the United States revealed that implicit, but not explicit maternal intuition confidence increased with baby age, and that implicit and explicit measures provided unique variance in predicting depressive symptomology, satisfaction with life, and maternal identity satisfaction. Study 2 provided an opportunity to replicate the associations of implicit maternal intuition confidence with satisfaction with life and maternal identity satisfaction in mothers from individualistic countries, and to extend the findings to examine mothers from collectivist countries. The positive association between implicit maternal intuition confidence and satisfaction with life was replicated across all countries, regardless of individualism or collectivism. In contrast, the positive association with maternal identity satisfaction was only replicated in mothers from individualist cultures.

Different findings of the influence of culture for well-being versus identity may point to both the universally distressing nature of feeling one doesn’t understand one’s baby and the culturally distinct processes that underlay identity development. Because members of individualist cultures derive their sense of self from internal attributes and feelings, it seems sensible that implicit maternal intuition confidence plays a role in how satisfied mothers from those cultures feel with their identity. In contrast, members of collectivist cultures derive their sense of self more strongly from others’ perceptions that they are fulfilling ascribed roles; it is possible that internal feeling states are not as important to maternal identity satisfaction, despite being important for overall life satisfaction. For mothers in collectivist cultures, identity may be scaffolded more upon external social aspects, such as being seen as adequate in the role by close others. In contrast, for mothers in individualist cultures identity may be more closely tied to self-feelings. This interpretation is speculative; these results must be replicated before we can be confident that cultural distinctions in the development of maternal identity satisfaction exist. Nonetheless, this work is the first, to our knowledge, to compare this aspect of Mercer’s (2004) model in such a wide sample of countries.

Whereas cultural differences in the association of maternal intuition confidence to identity satisfaction may reflect previously seen patterns in the importance of internal versus external aspects of identity (Markus and Kitayama, 1991), it is more difficult to speculate on the differences in maternal intuition confidence scores themselves. Although greater self-confidence is typically seen in individualist as compared to collectivist countries (Heine, 2005), specific confidence estimates for intuitive judgments are often higher in collectivist countries (Yates and de Oliveira, 2016). The differences observed here should be replicated. Additionally, because information on some cultural samples was limited (e.g. no information on racial background in Brazil), national differences should also be interpreted with caution.

Other limitations of the studies include their cross-sectional nature. The current findings suggesting a relationship between baby-age and implicit maternal intuition confidence, as well as associations of implicit maternal intuition confidence and well-being are both consistent with key aspects of Mercer’s (2004) maternal role attainment theory -namely that mothers grow increasingly more confident in their abilities to decipher the signals and needs of their infants across time, and this confidence undergirds satisfaction with motherhood. However, longitudinal studies examining the factors underlaying the growth of maternal intuition confidence (such as increased clarity of the baby’s communications) and alterations in maternal well-being and identity across time are needed to fully explore these dynamics. Additionally, the interplay between maternal intuition confidence (the feeling of knowing what one’s baby is thinking/feeling) and more behavioral competencies such as parental efficacy (confidence one can care for and soothe one’s baby) should be investigated as they grow over time, and as they relate to emotional well-being and identity satisfaction during the transition to parenthood. Parental efficacy has also been linked to depressive symptoms. However, unlike maternal intuition which is hypothesized to be developed anew with each individual child, parental efficacy has been found to increase with subsequent pregnancies (Leahy-Warren et al., 2012). Finally, it is likely that the relationship between maternal intuition confidence and well-being is bidirectional. Positive emotions both broaden attention and encourage flexible behavioral responses (Fredrickson, 2013), qualities potentially useful in developing maternal intuition confidence. It is thus possible that an upward spiral of maternal intuition confidence boosting well-being, and well-being in-turn boosting maternal intuition confidence could characterize the most positive transitions to motherhood.

Although implicit measures of maternal intuition confidence may be useful, in part, through bypassing unwillingness to report being less than an “ideal mother”(Mesman et al., 2015), we are not interpreting the modest correlation between implicit and explicit maternal intuition confidence as necessarily reflecting conscious deception. Implicit measures also tap into mental processes that individuals are unable (not just unwilling) to report, and it remains unclear how much the current measure reflects concerns less accessible to consciousness, as opposed to unreported due to social desirability. Future research examining the experiences and situations that support or threaten implicit maternal intuition confidence would provide valuable insight into this question. Additionally, future work using implicit measures to examine even more complicated and socially fraught maternal feelings (e.g. resentment toward their infant; Wittkowski et al., 2007) could allow novel examinations of factors underlying more negative experiences of parenting, such as burn-out.

Finally, the current research focused specifically on mothers, excluding fathers and other caregivers. In the majority of societies, mothers are the primary caregivers of infants and spend more time on infant-care than fathers even when both parents work outside the home (Raley et al., 2012). However, the study of parental intuition confidence should not be limited to mothers. Recent research has shown that, for fathers, the importance of parenthood to identity (Pew Research Center, 2015) and the prevalence of depression following the birth of an infant (DaCosta et al., 2019) are both increasing. Models of the transition to parenthood that were initially developed on mothers should be extended to include the experience of fathers – including examining the role of parental intuition confidence in well-being and identity.

Despite the limitations of the current work, results suggest a set of relatively simple and “low tech” decision confidence questions can reveal important psychological differences in maternal self-doubt vs. assurance. Due to relative ease of administration, this measure could be used in a broader range of environments (e.g. paper and pencil, verbal interview) than those that rely on response time or physiology. Implicit measures may be especially valuable when studying the transition to parenthood given both cognitive exhaustion and social desirability concerns that may prevent explicit self-report measures from capturing the full range of mothers’ experiences.

## Data Availability Statement

The datasets generated and all materials used for these studies are available by request from the corresponding author.

## Ethics Statement

All research was conducted in accordance with the ethical standards of the American Psychological Association. All participants gave written informed consent in accordance with the Declaration of Helsinki. The protocol for Study 1 was approved by the Institutional Review Board of Northwestern University, and for Study 2 by the Institutional Review Board of Integreview.com to ensure national ethics committee requirements for data collection in participating countries.

## Author Contributions

WG was responsible for data collection for Study 1, analyzed the data for Studies 1 and 2 and prepared the draft and final manuscript. KR and JN were responsible for overseeing data collection for Study 2 and provided feedback on analyses as well as on draft manuscripts. All authors provided critical feedback and helped shape the overall research.

## Conflict of Interest

WG has served as a consultant for Johnson & Johnson Consumer, Inc. KR and JN are employees of Johnson & Johnson Consumer, Inc.
